# Influence of Environmental Parameters on the Stability of the DNA Molecule

**DOI:** 10.3390/e23111446

**Published:** 2021-10-31

**Authors:** Alexander Svidlov, Mikhail Drobotenko, Alexander Basov, Eugeny Gerasimenko, Anna Elkina, Mikhail Baryshev, Yury Nechipurenko, Stepan Dzhimak

**Affiliations:** 1 Department of Radiophysics and Nanothechnology, Kuban State University, 350040 Krasnodar, Russia; svidlov@mail.ru (A.S.); mdrobotenko@mail.ru (M.D.); son_sunytch79@mail.ru (A.B.); anna013194@mail.ru (A.E.); baryshev_mg@mail.ru (M.B.); 2 Federal Research Center the Southern Scientific Center of the Russian Academy of Sciences, 344006 Rostov-on-Don, Russia; 3 Department of Fundamental and Clinical Biochemistry, Kuban State Medical University, 350063 Krasnodar, Russia; 4 Department of Technology of Fats, Cosmetics, Commodity Science, Processes and Devices Kuban State Technological University, 350072 Krasnodar, Russia; rosmaplus@gmail.com; 5 Engelhardt Institute of Molecular Biology, Russian Academy of Sciences, 119991 Moscow, Russia; nech99@mail.ru

**Keywords:** DNA, mathematical model, entropy, solvent viscosity, rotational movements of nitrogenous bases, dynamics of a double-stranded DNA molecule

## Abstract

Fluctuations in viscosity within the cell nucleus have wide limits. When a DNA molecule passes from the region of high viscosity values to the region of low values, open states, denaturation bubbles, and unweaving of DNA strands can occur. Stabilization of the molecule is provided by energy dissipation—dissipation due to interaction with the environment. Separate sections of a DNA molecule in a twisted state can experience supercoiling stress, which, among other things, is due to complex entropic effects caused by interaction with a solvent. In this work, based on the numerical solution of a mechanical mathematical model for the interferon alpha 17 gene and a fragment of the Drosophila gene, an analysis of the external environment viscosity influence on the dynamics of the DNA molecule and its stability was carried out. It has been shown that an increase in viscosity leads to a rapid stabilization of the angular vibrations of nitrogenous bases, while a decrease in viscosity changes the dynamics of DNA: the rate of change in the angular deviations of nitrogenous bases increases and the angular deformations of the DNA strands increase at each moment of time. These processes lead to DNA instability, which increases with time. Thus, the paper considers the influence of the external environment viscosity on the dissipation of the DNA nitrogenous bases’ vibrational motion energy. Additionally, the study on the basis of the described model of the molecular dynamics of physiological processes at different indicators of the rheological behavior of nucleoplasm will allow a deeper understanding of the processes of nonequilibrium physics of an active substance in a living cell to be obtained.

## 1. Introduction

Water is a universal solvent and medium in which most biochemical and biophysical reactions take place in the body. Depending on the chemical, biochemical, and even isotopic composition of the medium, the rate and nature of the biological processes occurring in it can change [1,2,3,4].

Thus, the reorganization of water molecules makes a significant contribution to changes in the enthalpy and entropy of DNA and proteins [5,6,7,8].

The free energy of the DNA nitrogenous bases pairs interactions [9,10,11,12] as well as the energy localization in it [13] depend on the parameters of the solvent. In addition, the dependence of the free energies of the nitrogenous bases stacking on the aqueous environment was noted [14].

It was found that the structure and dynamics of DNA can be influenced by water-miscible ethers of ethylene glycol through the implementation of mechanisms associated with hydrophobic catalysis. Experimental data show that this is accompanied by an increase in the energy of hydrogen bonds between pairs of nitrogenous bases and a decrease in the stacking energy [15].

Thus, it is obvious that it is necessary to consider biopolymers, taking into account the entropy and their interaction with the aqueous environment [16,17,18].

The study of the behavior of nucleic acids under the action of an external force at various indices of entropy in biological molecules is an actual topic [19,20]. It should be noted that almost all interactions of nucleoproteins and manipulations with DNA are associated with its mechanical deformations. Variations in the mechanical properties of DNA play a fundamental role in the regulation of various processes involved in the organization of chromatin on the scale of the entire genome [20], and DNA mechanics is an important component of its functional features [21,22]. Today, mechanical models are a powerful research method [23,24] that do not require a supercomputer to perform calculations. It should be noted that full atomic modeling is a separate complex task, for the solution of which it is necessary to take into account a huge number of parameters.

In our work, we investigated the mechanical properties of DNA using a mechanical mathematical model. The influence of the viscosity of the external environment on the internal dynamics and stability of the DNA molecule was investigated by the method of mathematical modeling.

## 2. Mathematical Model

To simulate the dynamics of a DNA molecule, we used a mathematical model that describes the rotational motion of nitrogenous bases around the sugar-phosphate chain. To build such a model, an analogy is used between a DNA molecule and a mechanical system consisting of two chains of interconnected pendulums.

In this case, the rotating pendulums corresponded to nitrogenous bases, and the elastic thread to which these pendulums are attached corresponded to the sugar-phosphate chains of the DNA molecule; the hydrogen bond of a pair of complementary nitrogenous bases corresponded to an elastic bond of the corresponding pair of pendulums [25].

This mathematical model includes the following Newton equations [26]:

(1) $$I_{1}^{i}\frac{d^{2}\varphi_{1}^{i}\left(t\right)}{dt^{2}}=K_{1}^{i}\left[\varphi_{1}^{i-1}\left(t\right)-2\varphi_{1}^{i}\left(t\right)+\varphi_{1}^{i+1}\left(t\right)\right]-\;-k_{12}^{i}R_{1}^{i}\left(R_{1}^{i}+R_{2}^{i}\right)\sin\varphi_{1}^{i}-k_{12}^{i}R_{1}^{i}R_{2}^{i}\sin\left(\varphi_{1}^{i}-\varphi_{2}^{i}\right)+\;+F_{1}^{i}\left(t\right),\;i=\bar{2,n-1},\left(1\right)$$

(2) $$I_{1}^{1}\frac{d^{2}\varphi_{1}^{1}\left(t\right)}{dt^{2}}=K_{1}^{1}\left[\varphi_{1}^{2}\left(t\right)-\varphi_{1}^{1}\left(t\right)\right]-\;-k_{12}^{1}R_{1}^{1}\left(R_{1}^{1}+R_{2}^{1}\right)\sin\varphi_{1}^{1}-k_{12}^{1}R_{1}^{1}R_{2}^{1}\sin\left(\varphi_{1}^{1}-\varphi_{2}^{1}\right)+\;+F_{1}^{1}\left(t\right),\left(2\right)$$

(3) $$I_{1}^{n}\frac{d^{2}\varphi_{1}^{n}\left(t\right)}{dt^{2}}=K_{1}^{n}\left[\varphi_{1}^{n-1}\left(t\right)-\varphi_{1}^{n}\left(t\right)\right]-\;-k_{12}^{n}R_{1}^{n}\left(R_{1}^{n}+R_{2}^{n}\right)\sin\varphi_{1}^{n}-k_{12}^{n}R_{1}^{n}R_{2}^{n}\sin\left(\varphi_{1}^{n}-\varphi_{2}^{n}\right)+\;+F_{1}^{n}\left(t\right),\left(3\right)$$

(4) $$I_{2}^{i}\frac{d^{2}\varphi_{2}^{i}\left(t\right)}{dt^{2}}=K_{2}^{i}\left[\varphi_{2}^{i-1}\left(t\right)-2\varphi_{2}^{i}\left(t\right)+\varphi_{2}^{i+1}\left(t\right)\right]+\;+k_{12}^{i}R_{2}^{i}\left(R_{1}^{i}+R_{2}^{i}\right)\sin\varphi_{2}^{i}-k_{12}^{i}R_{1}^{i}R_{2}^{i}\sin\left(\varphi_{2}^{i}-\varphi_{1}^{i}\right)+\;+F_{2}^{i}\left(t\right),\;i=\bar{2,n-1},\left(4\right)$$

(5) $$I_{2}^{1}\frac{d^{2}\varphi_{2}^{1}\left(t\right)}{dt^{2}}=K_{2}^{1}\left[\varphi_{2}^{2}\left(t\right)-\varphi_{2}^{1}\left(t\right)\right]+\;+k_{12}^{1}R_{2}^{1}\left(R_{1}^{1}+R_{2}^{1}\right)\sin\varphi_{2}^{1}1-k_{12}^{1}R_{1}^{1}R_{2}^{1}\sin\left(\varphi_{2}^{1}-\varphi_{1}^{1}\right)+\;+F_{2}^{1}\left(t\right),\left(5\right)$$

(6) $$I_{2}^{n}\frac{d^{2}\varphi_{2}^{n}\left(t\right)}{dt^{2}}=K_{2}^{n}\left[\varphi_{2}^{n-1}\left(t\right)-\varphi_{2}^{n}\left(t\right)\right]+\;+k_{12}^{n}R_{2}^{n}\left(R_{1}^{n}+R_{2}^{n}\right)\sin\varphi_{2}^{n}-k_{12}^{n}R_{1}^{n}R_{2}^{n}\sin\left(\varphi_{2}^{n}-\varphi_{1}^{n}\right)+\;+F_{2}^{n}\left(t\right).\left(6\right)$$

Here,

$\varphi_{j}^{i}\left(t\right)$—angular deviation of the *i*-pendulum of the *j*-chain, counted counterclockwise, at time *t*;

$I_{j}^{i}$—moment of inertia of the *i*-pendulum of the *j*-chain;

$R_{j}^{i}$—distance from the center of mass of the *i*-pendulum of the *j*-chain to the thread;

$K_{j}^{i}$—constant characterizing the torque of the *i*-section of the *j*-thread;

$k_{12}^{i}$—constant characterizing the elastic properties of the connection of the *i*-pair of pendulums (describes the elastic properties of hydrogen bonds between pairs of nitrogenous bases);

$F_{j}^{i}\left(t\right)$—external influence on the *i*-pendulum of the *j*-chain at time *t*; and

n—the number of pairs of pendulums in the system under consideration.

In Equations (1)–(6), the first term to the right of the equal sign describes the force action from the elastic thread on the i-pendulum; the second term is from the side of the paired pendulum; and the third term is the external force action. The magnitude of the external influence is taken equal to $F_{j}^{i}\left(t\right)=-\beta_{j}^{i}\frac{d\varphi_{j}^{i}}{dt}\left(t\right)+M\left(t\right)$, where the term $-\beta_{j}^{i}\frac{d\varphi_{j}^{i}}{dt}\left(t\right)$ models the effects of energy dissipation caused by the interaction with the liquid surrounding the DNA molecule, and the term M(t) models the external influence (the value of *M(t)* used by us coincides with the experimental [27]).

Note that the proposed model does not provide for the emergence of open states due to the breaking of hydrogen bonds.

We will add the initial conditions to Equations (1)–(6):

(7) $$\varphi_{1}^{i}\left(0\right)=\varphi_{1,0}^{i},\;\frac{d\varphi_{1}^{i}}{dt}\left(0\right)=\varphi_{1,1}^{i},$$

(8) $$\varphi_{2}^{i}\left(0\right)=\varphi_{2,0}^{i},\;\frac{d\varphi_{2}^{i}}{dt}\left(0\right)=\varphi_{2,1}^{i},\;i=\bar{1,n}.$$

For definiteness, we will assume that at t = 0, the system is in equilibrium, that is, in the initial conditions (7,8).

Problems (1)–(8) are the Cauchy problems for a system of 2n ordinary differential equations.

## 3. The Influence of the Viscosity of the External Environment on the DNA Dynamics

The influence of the external environment viscosity on DNA dynamics was investigated using the interferon alpha 17 gene (*n* = 980) and a fragment of the Drosophila gene (*n* = 5000) [28]. The values of the coefficients of Equations (1)–(6) were taken as presented in Table 1 (data are taken from [29]). At M(t) = 10^−22^ N·m, with this value of M(t), the pendulums’ angular deviation dynamics in the DNA molecule does not practically differ from the case of periodic external influences of the form cos(ωt)·10^−22^ N⋅m at ω ≤ 10^9^ s^−1^ [30]. The values of the coefficients $\beta_{j}^{i}$ in the right-hand side of Equations (1)–(6), characterizing the viscosity of the external medium, were equal to the corresponding values of β from Table 1, multiplied by the parameter λ. Thus, a change in the parameter λ corresponded to changes in the viscosity of the external medium (the λ parameter characterizing the viscosity of the surrounding liquid can depend on various factors: pressure, temperature, chemical composition). The parameter λ was taken from 0.1 to 4, since the viscosity of the environment can vary over a wide range [31].

The study of DNA dynamics was carried out on the basis of a numerical solution of problems (1)–(8), and the calculation results are presented in graphical form.

The dependence of the problem (1)–(8) solution from the viscosity was determined using the angular deviations and average angular deviations of the first chain of the DNA molecule:

(9) $$\varphi \left(t\right)=n^{-1}\overset{n}{\underset{i=1}{\sum }}\varphi_{1}^{i}\left(t\right)$$

**Figure 1:**
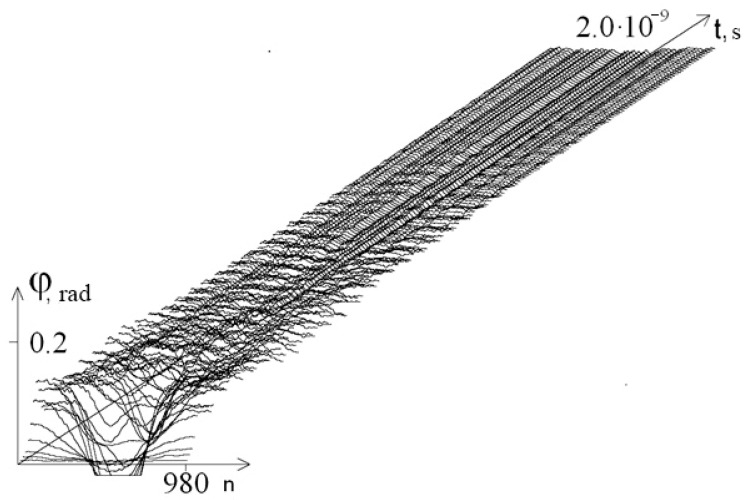
Graph of angular deviations of the first chain of the interferon gene in the interval [0; 2 × 10^−9^ s] at λ = 1.0.

Figure 1 shows a graph of the angular deviations of the first chain of the interferon gene in the interval [0; 2 × 10^−9^ s] at λ = 1.0.

From Figure 1, we see that after removing the system from equilibrium, a gradual stabilization of the amplitude of angular oscillations occurs.

**Figure 2:**
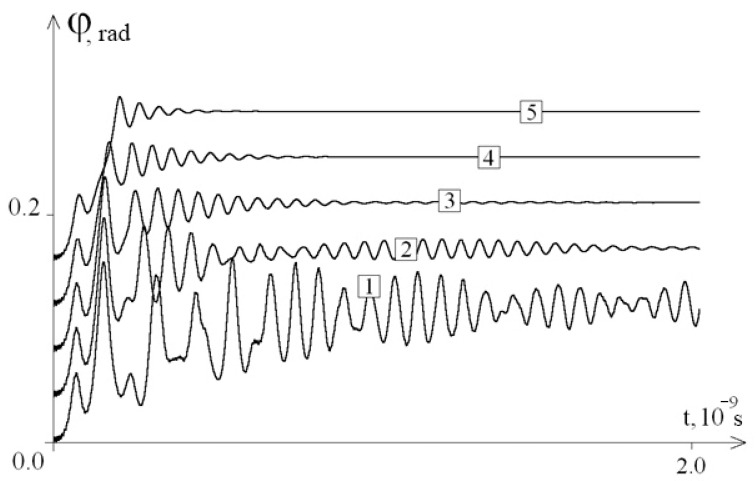
The average angular deviations (with vertical shift) of the first chain of the interferon gene in the interval [0; 2 × 10^−9^ s]: 1—at λ = 0.1; 2—at λ = 0.5; 3—at λ = 1.0; 4—at λ = 2.0; 5—at λ = 4.0.

Figure 2 shows the graphs of the average angular deviations of the first chain of the interferon gene in the interval [0; 2 × 10^−9^ s] for different values of parameter λ.

From Figure 2, we can see that a decrease in the viscosity of the external environment leads to an increase in the amplitude of the average angular deviations of nitrogenous bases, which can lead to the emergence of open states and unweaving of the DNA molecule.

**Figure 3:**
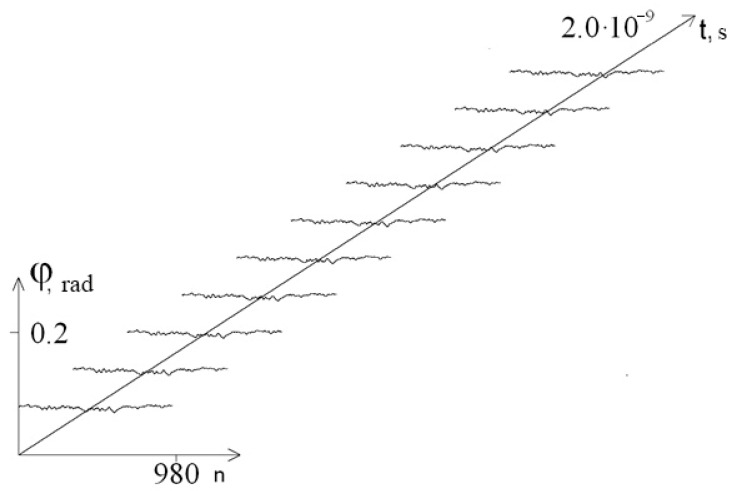
Graph of angular deviations of the first chain of the interferon gene in the interval [1.9 × 10^−9^ s; 2 × 10^−9^ s] at λ = 1.0.

**Figure 4:**
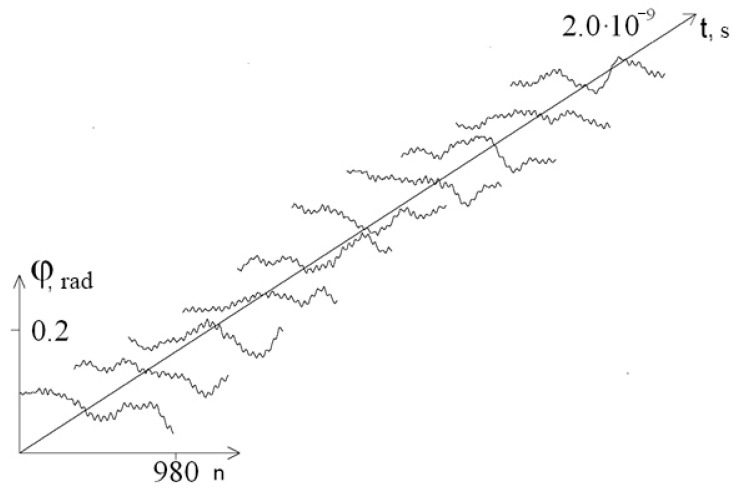
Graph of angular deviations of the first chain of the interferon gene in the interval [1.9 × 10^−9^ s; 2 × 10^−9^ s] at λ = 0.1.

Figure 3 and Figure 4 show graphs of the first chain angular deviations of the interferon gene in the interval [1.9 × 10^−9^ s; 2 × 10^−9^ s] at λ = 1.0 and λ = 0.1, respectively. It can be seen that at λ = 0.1, the graphs of the angular deviations change in time more significantly than at λ = 1.0 (which explains the increase in the amplitude of the mean angular deviations). In addition, at each moment of time at λ = 0.1, the DNA strand undergoes significantly larger angular deformations than at λ = 1.0, which leads to a decrease in the stability of the DNA molecule.

**Figure 5:**
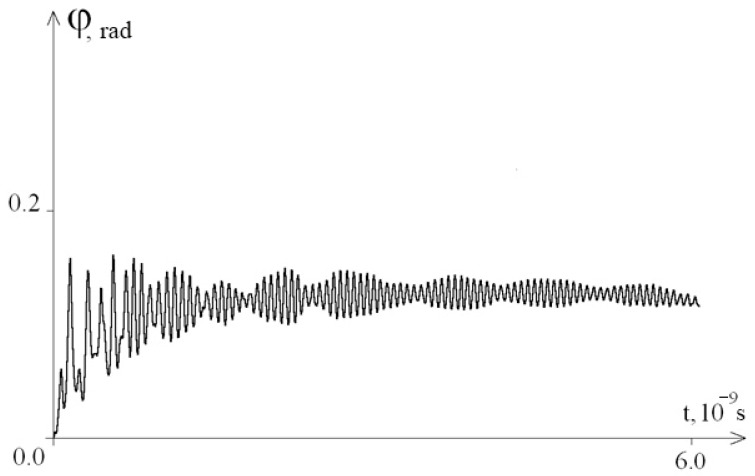
Graph of the average angular deviations of the first chain of the interferon gene in the interval [0; 6 × 10^−9^ s] at λ = 0.1.

**Figure 6:**
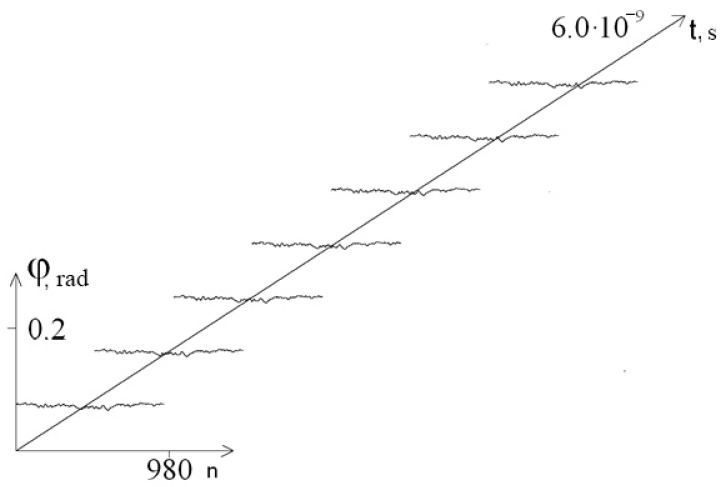
Graph of angular deviations of the first chain of the interferon gene in the interval [5.9 × 10^−9^ s; 6 × 10^−9^ s] at λ = 1.0.

**Figure 7:**
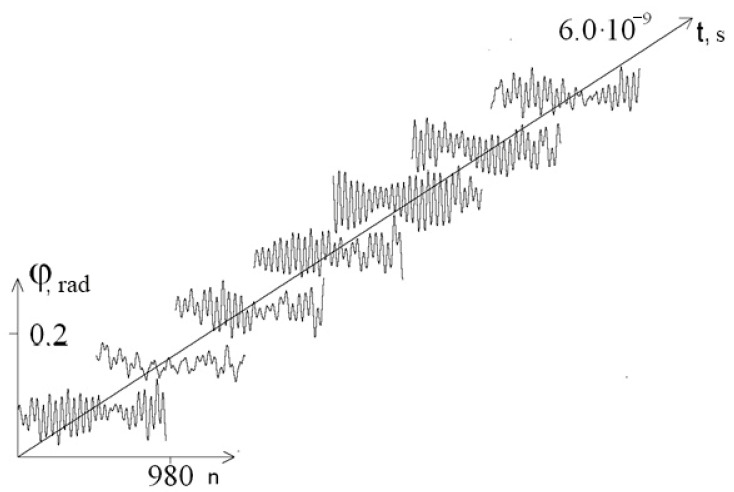
Graph of angular deviations of the first chain of the interferon gene in the interval [5.9 × 10^−9^ s; 6 × 10^−9^ s] at λ = 0.1.

For λ = 0.1, the time interval was increased to [0; 6 × 10^−9^s], and the results are shown in Figure 5 (graph of the average angular deviations of the first chain of the interferon gene presented); Figure 6 and Figure 7 show the graphs of the angular deviations of the first chain of the interferon gene in the interval [5.9 × 10^−9^ c; 6 × 10^−9^ s] at λ = 1 and λ = 0.1, respectively.

Figure 5, Figure 6 and Figure 7 show that over a long time interval at a low viscosity of the solvent (λ = 0.1), no attenuation of the amplitude of the angular deviations of nitrogenous bases was observed (a violation of the DNA molecule stability due to absence of vibration energy dissipation).

**Figure 8:**
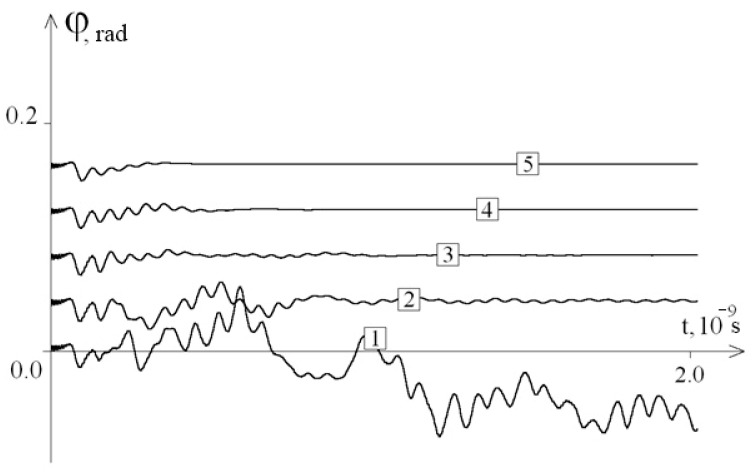
The average angular deviations (with vertical shift) of the first chain of the Drosophila gene fragment in the interval [0; 2 × 10^−9^ s]: 1—at λ = 0.1; 2—at λ = 0.5; 3—at λ = 1.0; 4—at λ = 2.0; 5—at λ = 4.0.

Figure 8 shows the graphs of the first chain average angular deviations of the Drosophila gene fragment (5000 bases) in the interval [0; 2 × 10^−9^ s] for different values of λ.

Figure 8 also shows that the dissipation of vibration energy due to the influence of the viscosity of the external environment on the dynamics of angular vibrations of the DNA molecule has the same characteristics as for the interferon gene.

**Figure 9:**
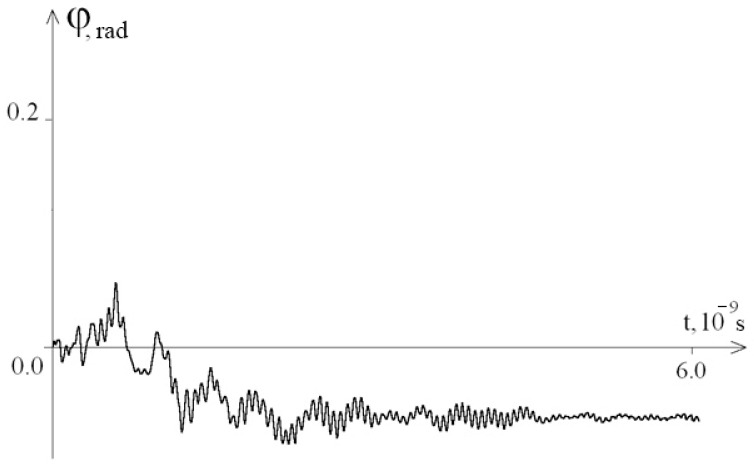
Graph of the average angular deviations of the first chain of the Drosophila gene fragment in the interval [0; 6 × 10^−9^ s] at λ = 0.1.

Figure 9 shows the graph of the average angular deviations of the first chain of the Drosophila gene fragment in the interval [0; 6 × 10^−9^ s] at λ = 0.1.

## 4. Discussion

It is known that the dynamics of a number of intracellular processes, primarily the transport of biomolecules and organelles within the nucleus as well as the peculiarities of the development of certain diseases, for example, Alzheimer’s and Parkinson’s, aging of the body, and various forms of cancer are accompanied by disturbances in the functioning of the genetic apparatus including the nucleolus, which was also characterized by pronounced changes in the rheological behavior of the nucleoplasm, one of the key mechanisms in the development of this pathology [32,33,34]. Using microrheological approaches, it has been shown in a number of works that the viscosity of the nucleoplasm, which refers to the content of the inner part of the nucleus outside the nucleolus, and represents the dissolved chromatin, ranges from 25 to 1000 Pa·s [35,36], significantly exceeding the viscosity of the nucleolus. Taking into account such wide fluctuations of viscosity inside the nucleus, the study of its influence on the appearance of open states is of particular interest, since when a DNA molecule passes from the region of high viscosity values to the region of its low values, it can lead to the emergence of open states [37], denaturation bubbles [38], and unweaving of DNA strands. Moreover, a large variability of angular deviations amplitude is typical for genes with a smaller nucleotide sequence (which can lead to an increase in DNA unweaving risk), and, consequently, failures in reading the genetic information, for example, due to destabilization of DNA supercoiling or violations of the torque of eukaryotic RNA polymerase. The described processes can be realized, for example, due to the influence of the viscosity of the DNA environment on the cooperative effects that are observed during the binding of low molecular weight ligands and regulatory proteins to DNA [39,40] and allosteric regulation of expression genes [41,42].

Molecule stabilization is provided by energy dissipation—dissipation due to interaction with the environment [43]. It should be noted that the hydration shell of the DNA molecule is inhomogeneous (both at the DNA–water interface and in the minor groove, where fluctuations in the groove width occur on the same time scale as the rearrangements of water hydrogen bonds) [44]. Separate sections of a DNA molecule in a twisted state can be under superhelical stress [45], which, among other things, is due to complex entropic effects caused by interaction with a solvent [9]. However, at this stage of the development of our model, we did not separate the phases of the aqueous environment, but considered all the water around the DNA molecule to be the same, which creates a general viscosity. The model takes into account the torsion of the sugar-phosphate strand, but the influence of the global conformation on the DNA dynamics is not taken into account. At the same time, the model makes it possible to take into account the effect of viscosity on any part of the DNA molecule.

Thus, the calculation results indicate the adequacy of the mechanical model used and the values of the coefficients. As noted earlier, mechanical models of DNA are a powerful tool for studying its properties [46]. At the same time, the mechanical model we used takes into account the heterogeneity of the sequence of nucleotide pairs, the energy of hydrogen bonds between pairs of nitrogenous bases, interaction with the environment, the appearance of open states [47], and makes it possible to replace a hydrogen atom with deuterium in hydrogen bonds between base pairs, etc. The model makes it possible to calculate the following parameters: the probability of occurrence of open states depending on the critical energy of hydrogen bonds; the likelihood of the appearance of bubbles of denaturation of various lengths throughout the gene [47]; to determine the most probable places of occurrence of open states in the gene [48]; and calculate the frequency of oscillations of a gene depending on external influences [30], etc.

## 5. Conclusions

In this work, on the basis of a numerical solution of a mechanical mathematical model for the interferon alpha 17 gene and a fragment of the Drosophila gene, an analysis of the influence of the viscosity of the external environment on the dynamics of the DNA molecule and its stability was carried out. It has been shown that an increase in viscosity leads to the rapid stabilization of angular vibrations, while a decrease in viscosity changes the dynamics of the DNA molecule: the rate of change in the angular deviations of nitrogenous bases increases and the angular deformations of the chains of the DNA molecule increase at each moment of time. These processes lead to instability of the DNA molecule, which increases with time. It is important to emphasize that the viscoelastic reorganization of the nucleoplasm, apparently, determines the rapid component of DNA relaxation inside the nucleus, supporting, for example, its supercoiling and preventing the occurrence of abnormal angular deviations that stimulate the emergence of open states. At the same time, genes with a smaller nucleotide sequence are characterized by a large amplitude of angular oscillations, which increases the risk of errors in reading information during transcription. Modeling the dynamics of a DNA molecule using the mathematical model presented in this work, is of interest to determine the risks of failures in reading genetic information in diseases accompanied by violations of microrheological parameters in the nucleus, which can be observed in various pathologies (neurodegenerative diseases of aging, oncology, and others). Additionally, the study on the basis of the described model of the molecular dynamics of physiological processes at different indicators of the rheological behavior of nucleoplasm will allow obtaining a deeper understanding of the processes of nonequilibrium physics of an active substance in a living cell.

## Figures and Tables

**Table 1:** Equation coefficients (1)–(6).

Type of Base	A	T	G	C
$I\cdot 10^{-44},\;\mathrm{kg}\cdot m^{2}$	7.61	4.86	8.22	4.11
R, Å	5.80	4.80	5.70	4.70
$K\cdot 10^{-18}$ , J	2.35	1.61	2.27	1.54
$k_{12}^{H}\cdot 10^{-2},\;N/m$	6.20	6.20	9.60	9.60
$\beta \cdot 10^{-34},\;J\cdot s$	4.25	2.91	4.10	2.79
